# Heat health risk assessment and identification of priority control areas in residential communities of Shijiazhuang

**DOI:** 10.3389/fpubh.2025.1624477

**Published:** 2025-07-16

**Authors:** Shikai Song, Yanrui Shang, Leibin Wang, Qiang Liu, Yuanjie Zhao

**Affiliations:** ^1^Postdoctoral Research Station of Geography, Hebei Normal University, Shijiazhuang, China; ^2^School of Geographical Sciences, Hebei Normal University, Shijiazhuang, China; ^3^Hebei Technology Innovation Center for Remote Sensing Identification of Environmental Change, Shijiazhuang, China

**Keywords:** Shijiazhuang, residential community, heat health risk, assessment, blue-greenspace

## Abstract

**Introduction:**

Under global warming, urban dwellers have been at significant health risk due to urban heat islands and frequent extreme heat events in recent years. Most previous assessments of heat health risk focused on the regional scale. Therefore, we aim to evaluate the fine level heat health risk of Shijiazhuang, China.

**Methods:**

Residential community was as choose as basic evaluation unit. The heat health risks of 1,086 residential communities in the main urban area of Shijiazhuang were assessed by the risk framework of the IPCC, in which the risk was multiplicatively aggregated by hazard, exposure, and vulnerability.

**Results:**

In 2023, the hazard followed a center-periphery pattern with decreasing value from the city center to the periphery, whereas vulnerability presented the opposite trend. This pattern aligned with the finding that hazard-dominant risk residential communities were generally distributed close to the center and the vulnerability-dominant risk appeared primarily near the periphery. Five villages some distance from the city center were evaluated to present very high risk, with vulnerability as the dominant risk factor. Two of the five villages were identified as priority control communities, and increasing the percentage of water bodies and vegetation was the most practical way to lower the heat health risk.

**Discussion:**

The differences in population exposure indicator may greatly affects the stability of heat risk mapping output. The results can assist urban managers in gathering comprehensive information about the heat health risk and developing effective mitigation strategies.

## 1 Introduction

Under the global warming and urban expansion, the growing population in cities faces the combined threats of frequent heatwaves and serious urban heat island effect in recent years (1–3). A precise and impartial evaluation of the heat health risk is a crucial prerequisite for the subsequent development of a mitigation plan and has recently become a hot research topic (1, 3–8). Current research mainly focused on administrative units or city block levels and lacked finer-scale exploration.

The first step in assessing the risk of heat risk is choosing an evaluation unit. An ideal assessment unit should be a geographical unit with low internal heterogeneity, be a governance unit easy for administrative management, and correspond with the spatial distribution of the evaluation data. Limited by accuracy of population data, prior heat-health assessments were mostly carried out at the administrative unit level using census data (3, 6, 8–11). Fortunately, gridded population data with a high spatial resolution of 100 to 200 m has been available in recent years, such as WorldPop and mobile signaling data (5, 12). These high spatial resolution data then contributed to risk assessments at the city block level, divided by roads, railway lines, lot boundaries, or waterways (13). Even at the block scale, there still exists the heterogeneity of population distribution, temperature, and land cover within the assessment unit (4).

To overcome the limitation of the administrative unit, the Local Climate Zone (LCZ) approach was employed (5, 14), and this approach is better suitable for study regions with significant differences in land use and cover between urban and rural areas. However, in urban areas, a zone may consist of multiple subdistricts or blocks, still resulting in heterogeneity, and does not match the governance unit.

Residential communities in cities are usually closed with regular internal elements and different landscapes outside, and considered to be the fundamental geographical unit for high-density population gathering. Different from city blocks, which include multiple types of land use and cover, such as communities, factories, commercial areas, forest and grass fields, and water bodies, residential communities are single functional residential land areas. In addition, residential communities are also considered as fundamental units under administrative and disaster emergency management. In the normal state, residential communities provide water and electricity for residents within the community, handle for cleaning and greening services, and implement government orders, while residential communities are responsible for population flow control and information transfer within a community under emergency conditions. Therefore, it is necessary to assess and analyze the heat health risk of residential communities, which were considered as ideal assessment units.

The selection of the assessment framework was the focus of further research after the evaluation unit was determined. There were three main frameworks in previous studies: the hazard-exposure framework for heat exposure assessment (9, 15), the exposure-sensitivity-adaptation framework for heat vulnerability assessment (11) and the hazard-exposure-vulnerability framework for heat health risk assessment (1, 3, 5, 8). Furthermore, two kinds of principles: additive (6, 8, 16) and multiplicative (1, 3, 5, 17), were used for aggregation of components in these frameworks.

Among the above three frameworks, hazard-exposure-vulnerability framework took Crichton’s risk triangle conceptual framework as the theoretical basis, which depicted hazard, exposure, and vulnerability as the three “sides” of the risk triangle (18). From a formal point of view, if any one component or “side” of the triangle was zero, then there was no risk under the multiplicative principle. However, unless all three elements were zero, the risk was not zero under the addition rule. Therefore, multiplicative aggregation can better reflect the relationships between risk and three elements than additive aggregation in the field of disaster risk research (5, 15, 19). Crichton’s risk triangle framework with multiplicative principle was employed and popularized by the Special Report on Managing the Risks of Extreme Events and Disasters to Advance Climate Change Adaptation of the IPCC (Intergovernmental Panel on Climate Change) (20) and its Fifth Assessment Report (21). Then this risk framework of IPCC has been widely applied in recent heat health risk assessment studies (1, 3, 5, 6, 22, 23) and the first comprehensive natural disaster risk survey in China during 2020 to 2022 (24). Consequently, this framework was used to guide the design of this study.

As the capital of Hebei Province, Shijiazhuang has experienced rapid industrialization and urbanization along with economic development, particularly over the last 20 years (25, 26). Extreme high-temperature events occurred frequently in Shijiazhuang (27). Especially in 2023, there were 39 days of daily maximum temperature above 35°C with the annual maximum temperature reaching 43.7°C based on temperature observation data. Besides, located east of the Taihang Mountains and west of the North China Plain, Shijiazhuang is a typical area where foehn occurs due to the large topographic drop (28).

However, there has been no heat health risks assessment about Shijiazhuang until now to our knowledge, despite its prominent extremely high temperature. Hence, this study takes residential communities in Shijiazhuang as the basic unit to assess the fine level heat health risk in 2023 based on the risk framework of IPCC, and the main objectives are: (i) to construct a heat health risk assessment framework; (ii) to assess, classify, and map risk and components; (iii) to detect the dominant index of risk; (iv) to identify the priority control communities. Results can provide a reference for the decision makers to accurately implement cooling measures.

## 2 Materials and methods

### 2.1 Study area and residential community

**Figure 1:**
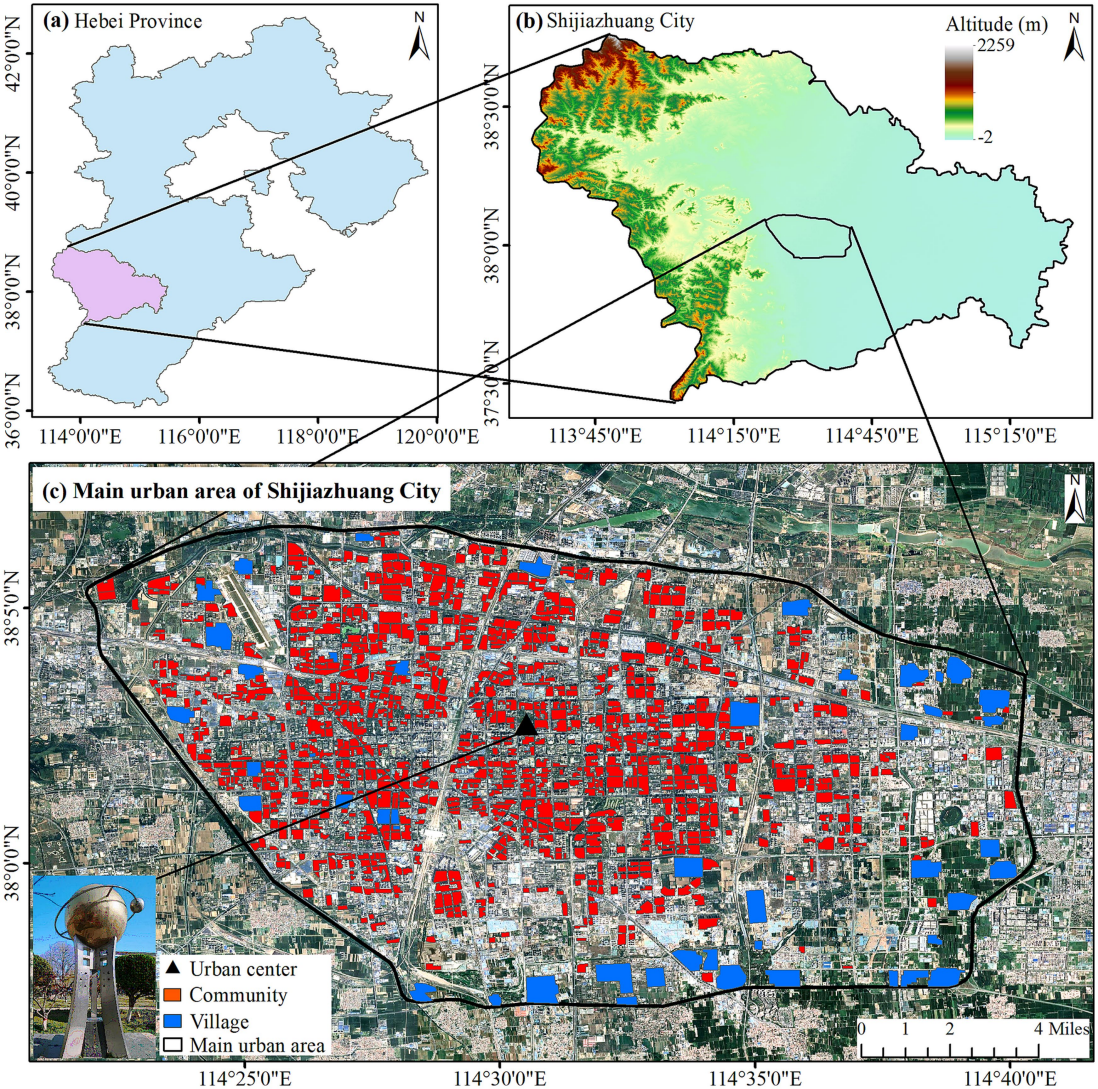
Study area.

Shijiazhuang, the capital city, is located in the southern region of Hebei Province (Figure 1a), with Taihang Mountains in the west part and Hebei Plain in the central and eastern parts (Figure 1b). The climate of Shijiazhuang is primarily a typical warm temperate continental monsoon climate with hot and rainy summer from June to August. Average monthly temperatures from June to August are 25.9, 27.1, and 25.6°C, with average monthly total precipitations of 53.0, 139.3, and 143.8 mm (26). The main urban area inside the Third Ring Road of Shijiazhuang was selected as the study area, covering a total area of 353.2 km^2^ (Figure 1c).

Residential communities in this study contain two types: community and village. A community contains groups of residential buildings with 6 to 35 floors and is usually enclosed by fences, while a village consists of houses built by the villagers themselves and is generally surrounded by cultivated land. Boundaries of residential communities were firstly obtained from Baidu map^1^ in 2023 then imported into ArcGIS. High resolution images from Google Earth in 2023 were used for further verification and an on-site inspection was conducted manually to determine the disputed boundaries. Finally, 1,037 communities and 49 villages were got as shown in Figure 1c. The minimum, maximum, and average areas of communities were 0.01, 0.29, and 0.06 km^2^, respectively, and villages with 0.02, 0.86, and 0.33 km^2^, respectively.

### 2.2 Heat health risk assessment framework

Our study employed the risk framework of IPCC for heat health risk assessment. In this well-established framework, the risk was multiplicatively aggregated by three fundamental components (hazard, exposure, and vulnerability), as Equation 1. Following previous literature (3, 5, 6, 16, 22), equal weights were set for three components since there is no widely accepted weighting at the present.


(1) Risk=Hazard×Exposure×Vulnerability

In the context of heat health risk assessment, hazard usually refers to high-temperature intensity and frequency (5, 15, 16). Exposure was described as the presence of people who could be adversely affected by high temperatures and heatwaves (1, 8). Vulnerability was measured by age and gender, socioeconomic status, and dwelling environment of people, which influence the capacity to anticipate, cope with, and resist high-temperature disasters (3, 5). Vulnerability emphasizes the features of residents and the internal attribution of the community (29). In this study, hazard was represented by land surface temperature due to data limitations. Population count rather than population density was considered as exposure. Vulnerability refers to the adaptability of residents to high temperatures, which is affected by the socioeconomic status of people, buildings features, and the community environment.

To quantify the three components, eight indicators of geographic, climatic socioeconomic, and demographic dimensions (Table 1) were determined after a thorough review of research (3, 5, 6) and refer to local geographical conditions of Shijiazhuang.

**Table 1:** Heat health risk assessment framework index system.

Target layer	Dimensional layer	Indicator layer	Indicator nature	Explanation of indicator	Weight
Risk	Hazard	Land surface temperature (LST) (°C)	Positive	Land surface temperature from Landsat images	0.64
		Percentage of impervious surface (PIS) (%)	Positive	The area ratio of impervious surface within a residential community	0.22
		Urban heat island effect (UHIE) (km)	Positive	The distance between a residential community and the city center	0.10
		Foehn effect (km)	Positive	The longitude difference between a residential community and the westernmost point of main urban area	0.04
	Exposure	Population count (people)	Positive	Total population count within a residential community	1
	Vulnerability	Housing price (yuan/m^2^)	Negative	Average housing price of a residential community	0.55
		Percentage of blue-greenspace (PBG) (%)	Negative	The area ratio of water body and vegetation within a residential community	0.45

It’s important to note that all the multi-source data were obtained in 2023 to keep the time consistent.

### 2.3 Indicators

#### 2.3.1 Land surface temperature

In the analysis of heat health risk, surface air temperature is more suitable to represent hazards than land surface temperature (LST) (8), but the sparse and uneven distribution of weather stations cannot meet the requirements of spatial analysis about complex changes in urban heatwaves. Therefore, this study followed previous studies (1, 6, 10, 15) to use LST from remote sense images as an indicator for hazard.

LST images were retrieved from Landsat 8.0 and Landsat 9.0 through Google Earth Engine. Image selection met the following conditions: The imaging date was within summer (June to August) in 2023; The weather of the day with priority was clear, with calm wind, no clouds or less clouds; Daily maximum temperature exceeding 35°C. Finally, images of May 15th and August 3rd from Landsat 8.0 and June 24th from Landsat 9.0 were collected, and daily maximum temperatures were 36, 36, and 39°C, respectively. The final LST image was averaged by selected images to minimize the data bias. Then the pixels of the final LST image within the residential community boundary and except ones on building roofs were extracted, and the values of pixels were averaged to be the LST for each community.

#### 2.3.2 Land cover

**Figure 2:**
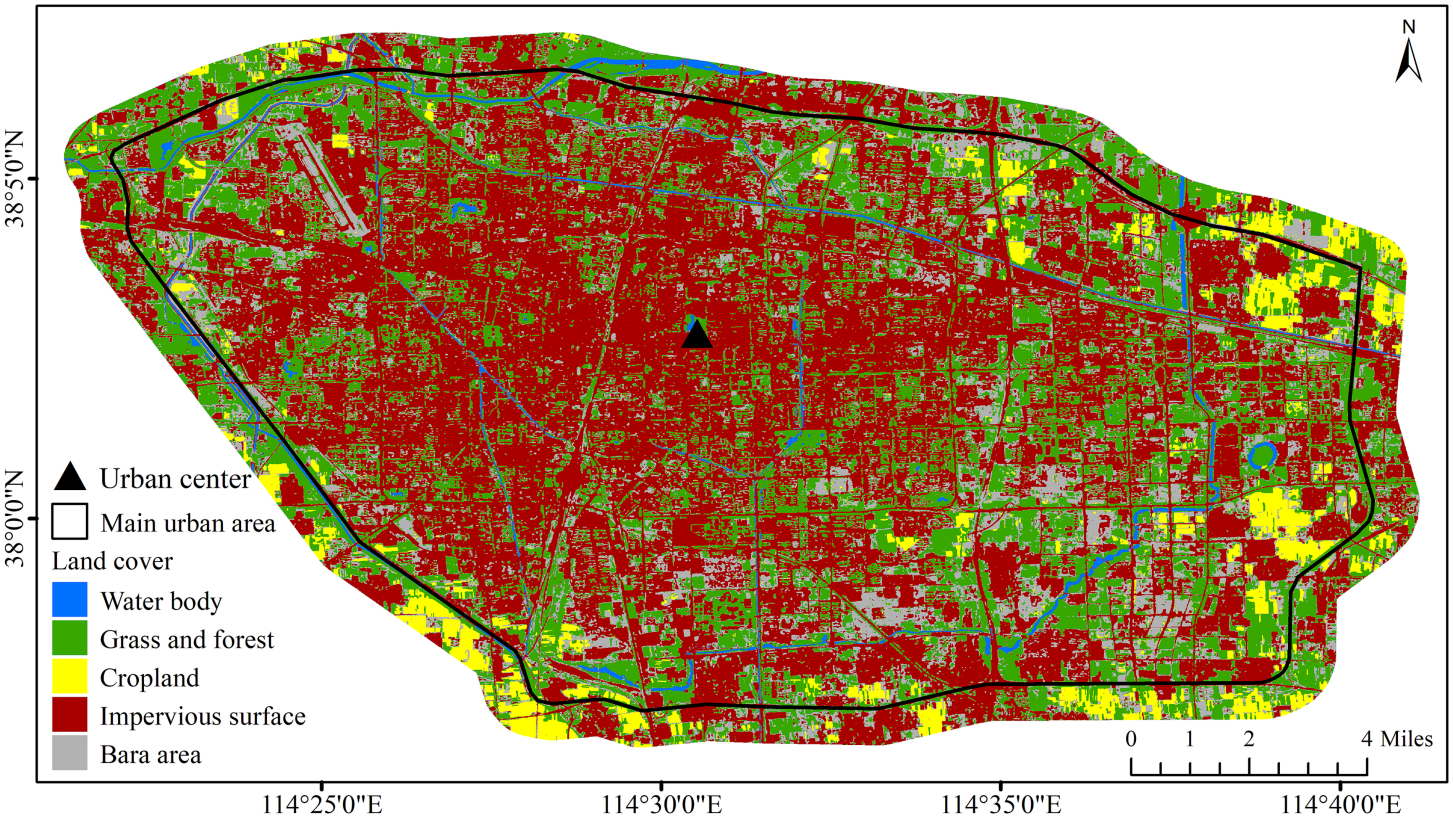
Land cover map.

Multi-temporal Sentinel-2 images with a spatial resolution of 10 m in 2023 were classified by random forest classifier to generate the land cover map, using the method employed in the study of (26). The classification result with an overall accuracy of 84.2% is displayed in Figure 2. Then the classification map was extracted by the residential community layer to get land cover for each community.

According to the surface temperature characteristics of land cover, the percentage of impervious surface (PIS) within a residential community was chosen as an indicator of hazard and the percentage of blue-greenspace (PBG) as an indicator of vulnerability to reflect the cooling adaptability of a residential community.

#### 2.3.3 Urban heat island and foehn effect

Shijiazhuang coordinate system reference point is located in Changan Park, less than 200 meters away from the mass center of 1,086 residential communities, regarded as the city center (Figure 1). Generally speaking, the city center typically experiences warmer temperatures than the surrounding suburbs in a city with single core (30). Hence, urban heat island effect (UHIE) was selected as the second indicator of hazard by reference to (8), and the indicator value was the reciprocal of the distance between the residential community and the center point (abbreviated as “the Distance” below).

Foehn winds with heat from the Taihang Mountains in the west generally blow to the main urban area in the east, and the temperature is higher in the west of Shijiazhuang (28). The third indicator of hazard was therefore the foehn effect, which is the reciprocal of the longitude difference between the residential community and the westernmost point of the main urban area.

#### 2.3.4 Population

The WorldPop data has been used in heat health risk assessment due to its high spatial resolution (5, 31); however, it only has data prior to 2021, which is incongruous with other 2023 data presented in this research. The mobile signaling data has high timeliness besides a fine spatial resolution (12, 15), and consequently was employed in this paper as a proxy of population exposure.

Hourly mobile phone signaling data with 160 m × 160 m resolution in October 2023 were acquired from China Mobile. 6 days of National Day holiday and 6 days of weekend were taken away because of the unconventional mobility of the population. Most people get off work at about six o’clock pm, stay at home after eight, and turn off phones after 10 based on the local schedule. As a result, mobile phone signaling data from 8 to 10 o’clock pm in 19 days was averaged as population data, covering 6,209 spatial grids and 2,712,636 people. Then the averaged data was intersected with the residential community layer to get value for each residential community, and the total population of 1,086 residential communities was 2,006,169, accounting for 73.96% of the population in the main urban area.

#### 2.3.5 Housing price

In addition to PBG, housing price as one indicator of vulnerability was derived from the Anjuke Real Estate Network^2^ in 2023. Housing price was introduced to reflect the economic conditions and the ability of residents to cope with the challenge caused by high temperatures indoor (11). Residents with higher economic levels usually can afford more heat reduction equipment, medical institutions, and infrastructure to mitigate extreme heat (3).

### 2.4 Data processing and calculation

#### 2.4.1 Indicators normalization

To eliminate differences in the magnitude, scale, and attribution, all indicators (five positive and two negative indicators) were normalized to a dimensionless value ranging 0.1 and 0.9 using Equation 2 proposed by (32) to avoid the presence of zero values in the results.


(2) $$V^{\prime }=0.1+\{\begin{array}{c} \frac{V-\min}{\max-\min}\times \left(0.9-0.1\right),\mathrm{Positive indicators}\\ \frac{\max-V}{\max-\min}\times \left(0.9-0.1\right),\mathrm{Negative indicators} \end{array}$$

Where *V*′ is the standardized value, *V* is the original value, and *Min* and *Max* represent the minimum and maximum values of the original indicators, respectively.

#### 2.4.2 Indicator weights and aggregation for risk component

Drawing on the method in (1), the relative weights of hazard and vulnerability indicators were determined based on an analytic hierarchy process pairwise comparison survey. Eleven survey questionnaires administered by experts were collected, and weights of indicators were shown in Table 1. Hazard and vulnerability were aggregated by standardized indicators as Equation 3.


(3) $$\mathrm{Component}=\sum_{i=1}^{j}\;\left({\mathrm{Standardized indicator}}_{i}\times {\mathrm{Weight}}_{i}\right)$$

#### 2.4.3 Multiplication of components for risk

Then the three components were normalized by Equation 2 and multiplied together with equal weights to get the risk by Equation 1.

#### 2.4.4 Classification into ranks

Finally, indicators, components, and risk were categorized into five ranks (very low, low, medium, high, and very high) using the equal breaks method. The majority of previous research generally preferred Jenks natural breaks method for classification in the risk assessment field (5, 11). However, equal breaks are more suitable for comparing the rank distribution of various data (1, 6).

### 2.5 Identification of priority control community

When a high-temperature disaster occurs, timely medical treatment is the primary measure for heatstroke patients to relieve pain (22). Long distances to healthcare services decrease the possibility of obtaining efficient medical treatment (6). Hospitals outside of the community can represent society’s coping capacity to heat-stroke patients, in contrast to vulnerability, which emphasizes the adaptability of residents to extreme heat, reflected by characteristics of residents and internal attribute of the community. In order to create more precise prevention and control strategies, priority control communities could be identified by combining the location of general hospitals with the findings of heat health risk assessment drawing on the research thought of (3). Thirty-five general hospitals within the main urban area were obtained from Baidu Map^3^ in 2023.

## 3 Results

### 3.1 Characteristics of indicators

**Figure 3:**
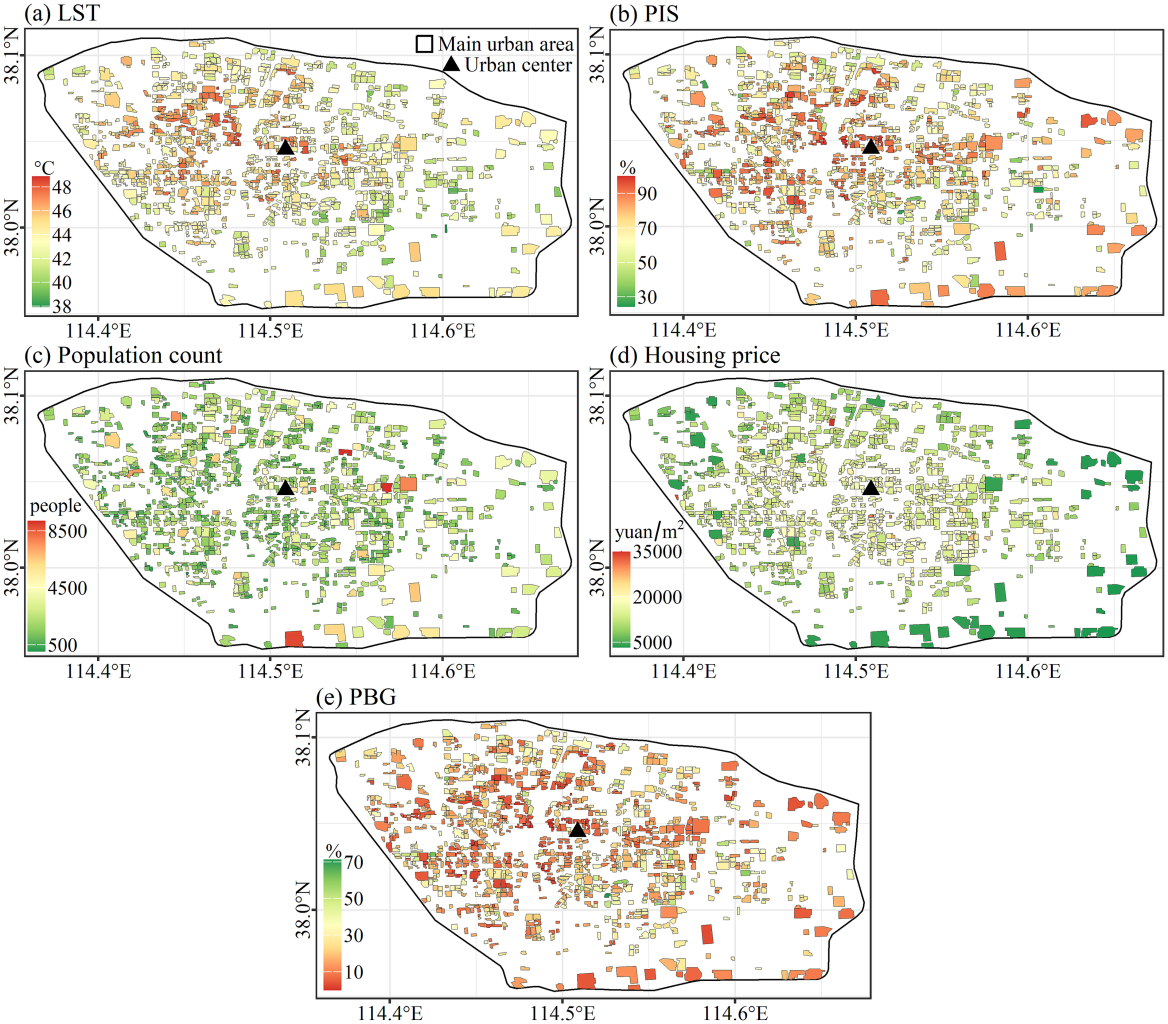
Spatial distribution of indicators.

**Figure 4:**
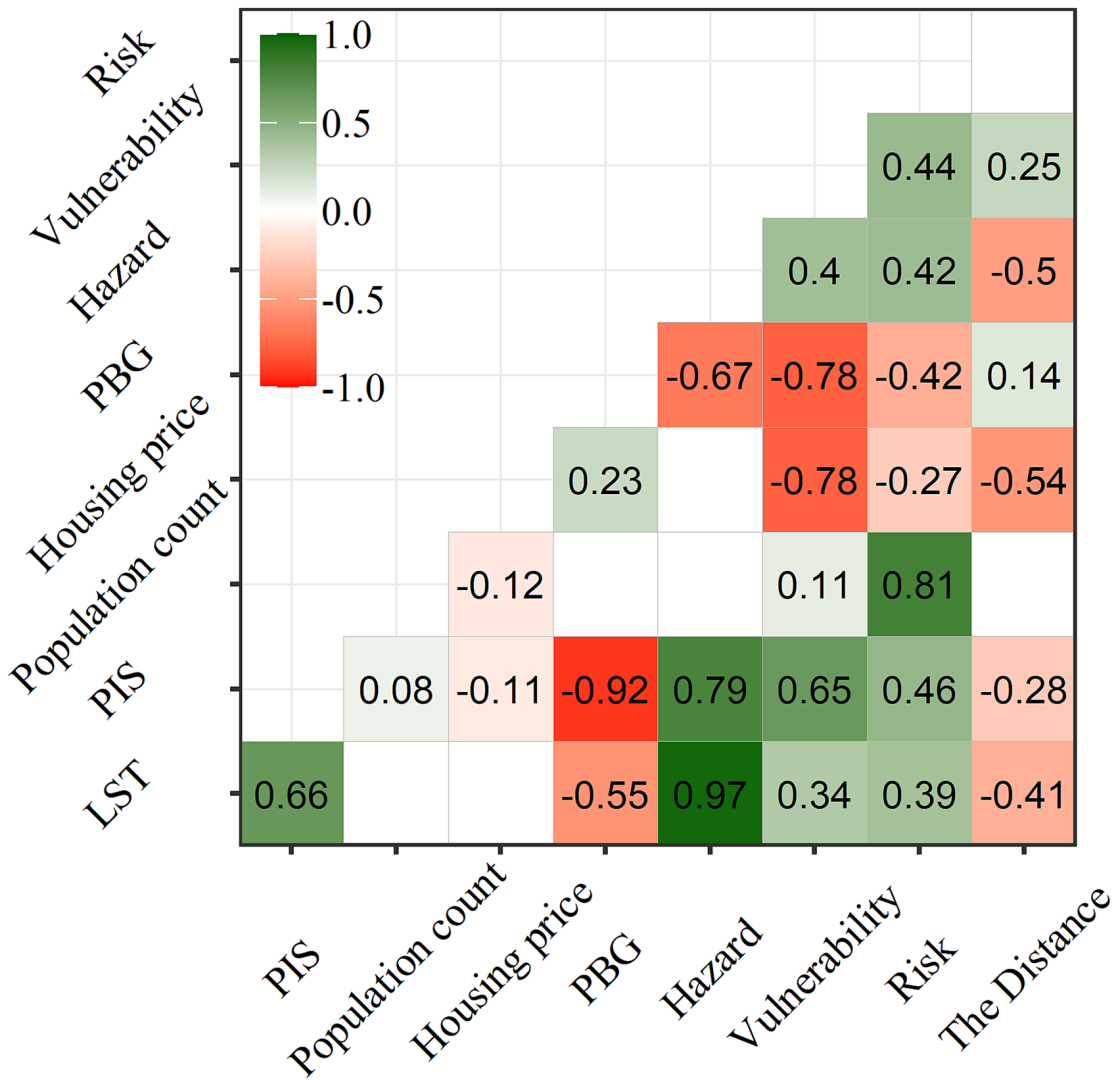
Correlation matrix among indicators.

The spatial distribution of five indicators of 1,086 residential communities is shown in Figures 3, 4 displays the correlation matrix among these indicators. The correlation between the indicators and the Distance revealed that LST, PIS, and housing price showed a center-periphery spatial pattern with decreasing trend (*R* = −0.41, −0.28, and −0.54), while PBG increased from center to periphery (*R* = 0.14). As for population count, its spatial distribution was irregular and did not show a center-periphery pattern (Figures 3, 4).

The minimum, maximum, and average values of five indicators about two types of residential communities were presented in Table 2. In comparison to villages, the community’s average LST, PIS, and population count were lower at 43.9°C, 72.6%, and 1786 people, respectively, compared to 44.5°C, 82.0%, and 3,253 people, respectively. However, the average housing price and PBG of the community (13,934.0 yuan and 19.1%) were higher than the village (3816.9 yuan and 10.3%).

**Table 2:** Minimum, maximum, and average value of five indicators.

Indicator		Minimum value	Maximum value	Average value
Community	LST (°C)	37.9	48.9	43.9
	PIS (%)	24.3	100.0	72.6
	Population count (people)	21	9,170	1786
	Housing price (yuan/m^2^)	5,344.0	35,000.0	13,934.0
	PBG (%)	0.0	71.3	19.1
Village	LST (°C)	41.2	48.1	44.5
	PIS (%)	40.1	98.0	82.0
	Population count (people)	217	8,837	3,253
	Housing price (yuan/m^2^)	3259.2	4416.1	3816.9
	PBG (%)	1.8	35.2	10.3

### 3.2 Characteristics of three components and risk

**Figure 5:**
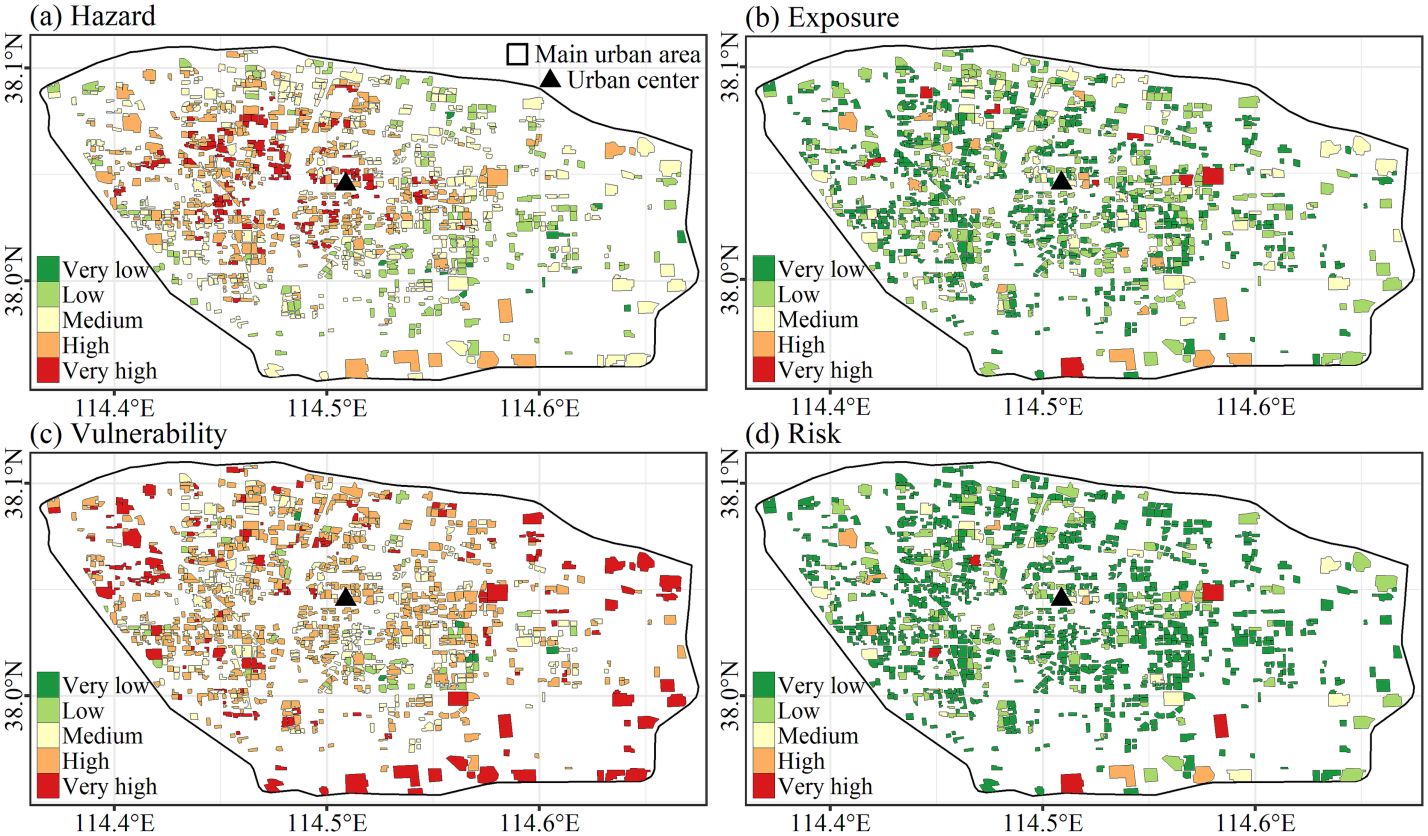
Spatial distribution of hazard, exposure, vulnerability, and risk.

Figure 4 indicated that both the LST and PIS decreased with the Distance. As the weighted sum of LST and PIS, hazard accordingly followed this decreasing trend (*R* = −0.5). The spatial distribution of vulnerability also presents this center-periphery pattern, but the trend was low inside and high outside (*R* = 0.25), which was opposite to the hazard. The spatial distribution of exposure (population count) was irregular and did not show a center-periphery pattern (Figures 4, 5).

**Figure 6:**
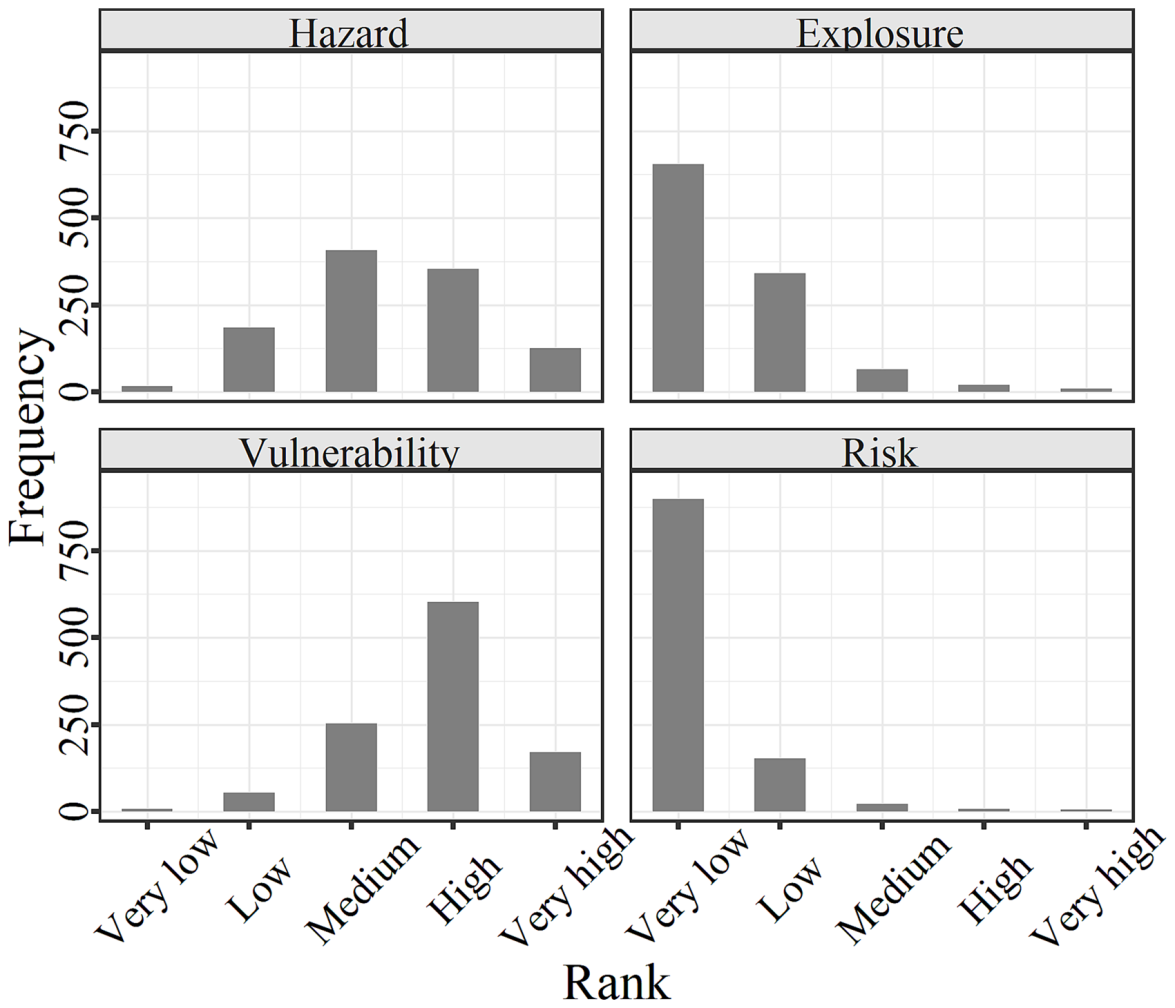
Frequency distribution of classified hazard, exposure, vulnerability, and risk.

The frequency distribution histogram of classified hazard, exposure, vulnerability, and risk is shown in Figure 5. With regards to hazard and vulnerability, medium and high rank was more than low and very high rank, followed by very low rank. Majority of exposure ranks were very low, with low, medium, high, and very high-rank frequencies decreasing in turn (Figure 6).

In terms of risk, as the product of hazard, exposure, and vulnerability, the risk highly correlated to hazard (*R* = 0.42), exposure (*R* = 0.81), and vulnerability (*R* = 0.44) (Figure 4). The opposite spatial distribution of hazard and vulnerability, combined by the irregular pattern of exposure resulted in randomization for spatial distribution of risk (Figures 4, 5). Only five residential communities were very high, with 7, 18, 133, and 941 residential communities at high, medium, low, and very low rank, respectively, which was similar to that of exposure (Figure 6).

### 3.3 Dominant factor of risk

**Figure 7:**
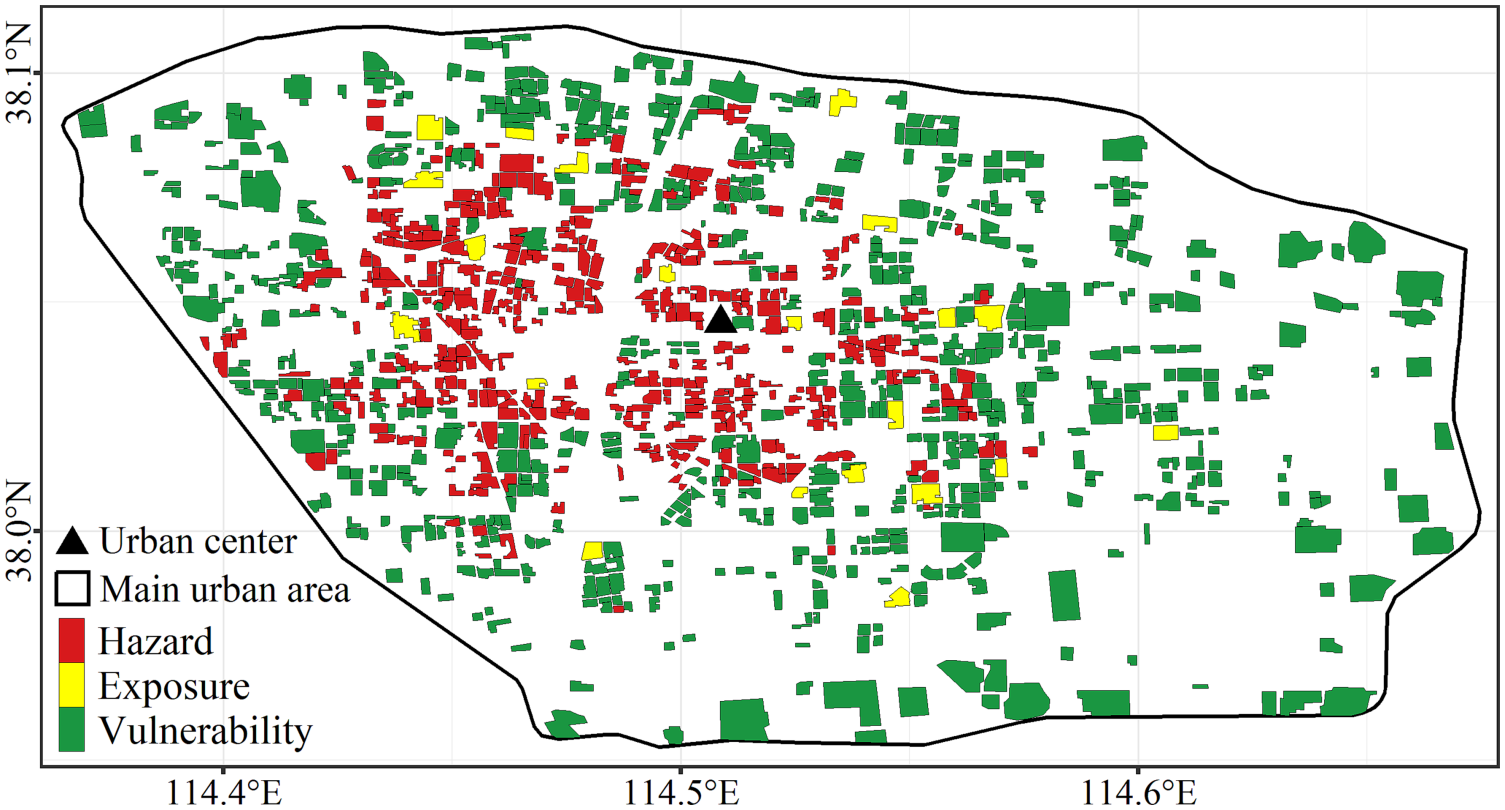
Spatial distribution of dominant factor of risk.

Normalized hazard, exposure, and vulnerability of each residential community were compared, and the maximum value was considered as the dominant factor of risk. Hazard and vulnerability-dominant type accounted for 46.3 and 50.8%, with exposure-dominant type only for 2.9% of the total 1,086 residential communities (Figure 7). Hazard-dominant residential communities were mainly concentrated near the urban center, while vulnerability-dominant residential communities appeared primarily close to the periphery. This finding aligns with the observation shown in Figure 5 that hazard and vulnerability present a center-periphery pattern with high hazard and low vulnerability inside and low hazard and high vulnerability outside.

### 3.4 Very high-risk residential communities and priority control identification

#### 3.4.1 Very high-risk residential communities

**Figure 8:**
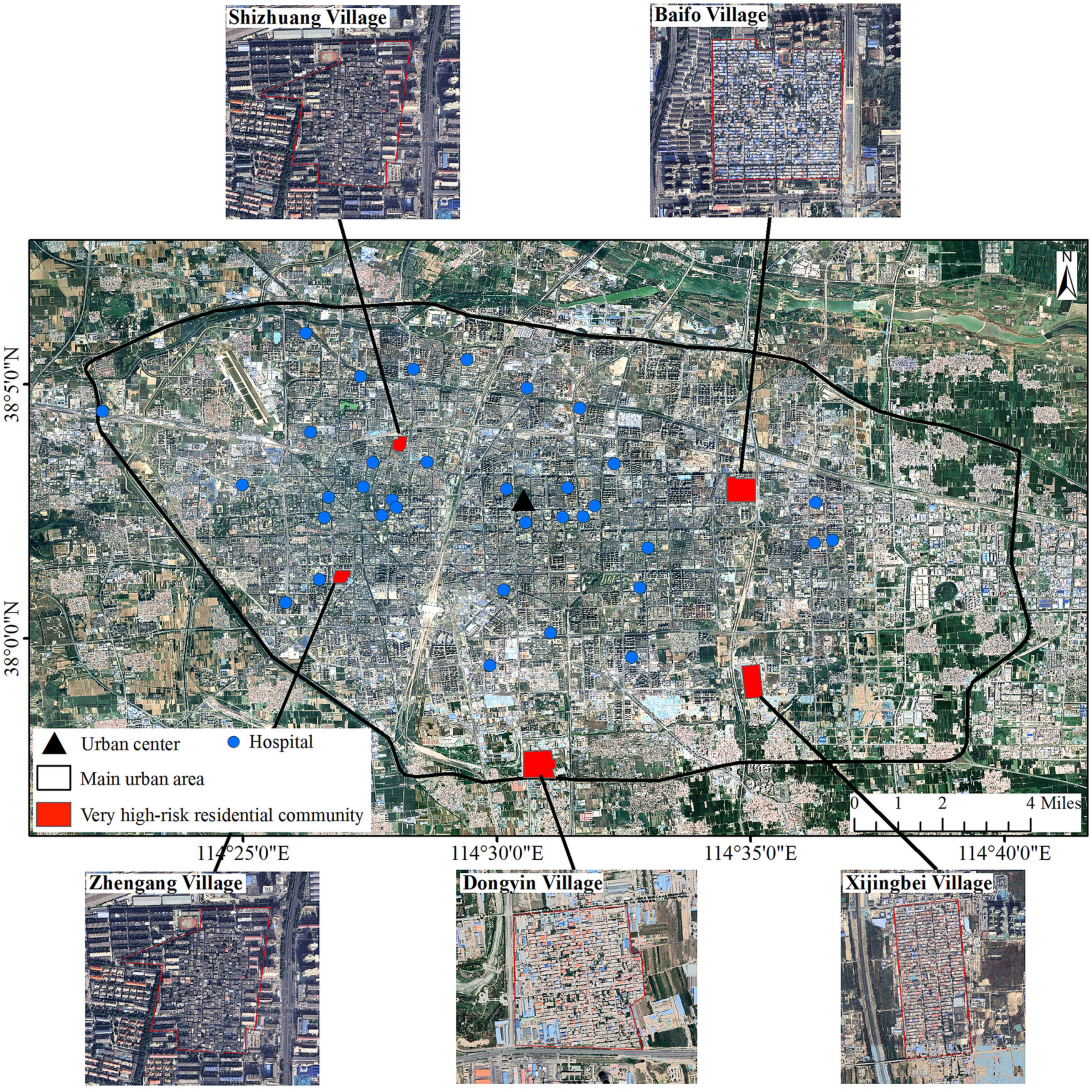
Spatial distribution and images of very high-risk residential communities.

Attributes and locations of five residential communities with very high risk are presented in Table 3 and Figure 8. All five residential communities were village type with some distances away from the city center, named Dongyin Village, Shizhuang Village, Zhengang Village, Baifo Village, and Xijingbei Village.

**Table 3:** Attributes and ranks of very high-risk residential communities.

Name	Type	LST	PIS	UHIE	Foehn effect	Hazard	Population count	Population density	Housing price	PBG	Vulnerability	Risk	Dominant factor	Distance to hospital (km)
Dongyin	Village	4	5	3	2	4	5	1	1	1	5	5	Vulnerability	3,272
Shizhuang	Village	5	5	2	3	5	4	2	1	1	5	5	Vulnerability	652
Zhengang	Village	4	5	2	3	4	4	2	1	1	5	5	Vulnerability	409
Baifo	Village	4	5	3	1	4	5	1	1	1	5	5	Vulnerability	1722
Xijingbei	Village	4	5	4	1	4	4	1	1	1	5	5	Vulnerability	3,189

According to the Google images (Figure 8), all these villages have a high density of housing, low vegetation coverage, and no water bodies, resulting in very high PIS and very PBG (Table 3). The hazard and exposure of the five villages were high or very high. All the vulnerabilities were very high and the dominant factor in risk for the five villages (Table 3).

#### 3.4.2 Priority control identification and control strategy

According to the distance between the five villages and the nearest hospital (Figure 8; Table 3), Dongyin Village and Xijingbei Village were the most distant from the hospital and were identified as priority control communities.

The control strategy should be developed following the identification of the priority control communities. Reducing vulnerability was considered as the primary objective since vulnerability was the dominant factor of risk for both Dongyin Village and Xijingbei Village. The correlation coefficient (*R* = 0.23) indicates that increasing IBG could raise housing prices, which jointly helped to reduce vulnerability (*R* = −0.67 and −0.73). Additionally, the expansion of blue and green space also could lower LST (*R* = −0.57), then decrease hazard (*R* = −0.67) (Figure 4). Besides, it was difficult to adjust other seven indicators in three components of risk. As a result, increasing water bodies and vegetation space was currently the best measure to reduce heat health risk for Dongyin Village and Xijingbei Village. In addition, new hospitals should be built around to improve coping capacity.

## 4 Discussion

### 4.1 Consistent of data time

The heat health risk assessment is a comprehensive work involving multiple types and dimensions of data, such as meteorological and climate, population age and structure, land use and cover, social economy, medical treatment, public facilities, etc. (1, 3, 5, 6). Due to the difficulty of data acquisition, some studies ignored the consistent of data time, which usually leads to uncertainty about results and lack of comparison with other research (15). To avoid such a defect, all the data in this study were obtained within 2023, but there were still seasonality differences in exposure and hazard data. Mismatch between LST from summer and mobile signaling data from October may lead to both low population exposure and low risk.

### 4.2 Population data

Although population density has been employed as an exposure indicator in other studies (3, 5, 8, 15, 16), exposure in this study refers to population count within each residential community. Water and electricity supply takes the community as the basic unit, and the more people, the greater the supply pressure under heat waves. Therefore, population count was more suitable than population density for heat-health assessments based on residential communities.

However, using mobile phone signaling data may have caused some uncertainty in the results and inconsistencies with other studies. It is widely acknowledged that vulnerable groups—such as children, the older adult, pregnant women, and people with disabilities—are more susceptible to the negative effects of heat (6, 8). The percentage of vulnerable groups is usually regarded as one of the indicators of vulnerability in previous studies. The mobile signaling data used in this paper lacks gender and age attributes because of the limitation of data access permission. In the future, if there are detailed population survey data for household visits, precise vulnerability estimation may be carried out.

### 4.3 Characteristics of very high-risk residential communities

Based on the assessment results of the entire region, key areas or hotspots need to be further confirmed (13, 33). In this study, heat health risks of 1,086 residential communities in 2023 over the main urban area of Shijiazhuang were evaluated, and five villages were evaluated as very high-risk communities. We found that these five villages were some distances away from the city center, and vulnerability was the dominant factor of risk. This finding was inconsistent with some studies (3, 6, 16). Hua (16) considered that high population exposure was a main contributor to the high-risk level of most hot spots. Wang (6) identified high heat risk areas appearing primarily in higher-density residential areas. Su (3) reported that inner-city residents were at a higher heat health risk compared to residents of other districts mainly owing to high temperature and population exposure.

Above inconsistencies may be due to differences in population exposure indicators. All the population count of five very high-risk villages was very high, while population density was at low and very low levels (Table 3), which will result in different risk values. Zhang (22) compared four population indicators: number, proportion, built-up population density, and total population density, then also found that the differences in population exposure indicators greatly affects the stability of heat risk mapping output.

## 5 Conclusion

This study takes residential communities in Shijiazhuang as the basic unit to assess the fine level heat health risk in 2023 based on the risk framework of IPCC. The conclusions are as follows:

1. Spatial distribution of LST, PIS, housing price, and PBG showed a center-periphery pattern. Hazard and vulnerability also followed this circular structure with decreasing hazard and increasing vulnerability from the city center to the periphery. Five residential communities with some distances away from the city center were evaluated at very high risk, while most residential communities were classified into very low levels.
2. There were two main types of risk dominant factor: vulnerability-dominant type for 552 residential communities that were primarily distributed close to the periphery and hazard-dominant type for 503 residential communities that mainly gathered near the urban center. This distribution resulted from the opposite trend of the center-periphery pattern for hazard and vulnerability.
3. All the five very high-risk residential communities were villages with a high density of housing, leading to high or very high hazard and exposure, and very high vulnerability. Furthermore, vulnerability was the dominant factor for the five villages. According to the distance of the nearest hospital, Dongyin Village and Xijingbei Village were identified as priority control communities.
4. For priority control communities, increasing vegetation and water bodies is now the most practical way to lower the heat health risk. Additionally, new hospitals should be built around to improve coping capacity.

This study can contribute to the foundation work for prevention and control of urban heat disasters in Shijiazhuang. In our future work, we will try to analyze the current situation of shelters in the context of extreme heat waves and provide rational layout strategies.

## Data Availability

The original contributions presented in the study are included in the article/supplementary material, further inquiries can be directed to the corresponding author.

## Footnotes

^1^https://map.baidu.com/

^2^https://sjz.anjuke.com/

^3^https://map.baidu.com/
